# Genomic diversity of the human intestinal parasite *Entamoeba histolytica*

**DOI:** 10.1186/gb-2012-13-5-r38

**Published:** 2012-05-25

**Authors:** Gareth D Weedall, C Graham Clark, Pia Koldkjaer, Suzanne Kay, Iris Bruchhaus, Egbert Tannich, Steve Paterson, Neil Hall

**Affiliations:** 1 Institute of Integrative Biology, University of Liverpool, Biosciences building, Crown Street, Liverpool L69 7AH, UK; 2 Pathogen Molecular Biology Department, Faculty of Infectious and Tropical Diseases, London School of Hygiene and Tropical Medicine, Keppel Street, London WC1E 7HT, UK; 3 Bernhard Nocht Institute for Tropical Medicine, Bernhard-Nocht-Str. 74, 20359 Hamburg, Germany

## Abstract

**Background:**

*Entamoeba histolytica *is a significant cause of disease worldwide. However, little is known about the genetic diversity of the parasite. We re-sequenced the genomes of ten laboratory cultured lines of the eukaryotic pathogen *Entamoeba histolytica *in order to develop a picture of genetic diversity across the genome.

**Results:**

The extreme nucleotide composition bias and repetitiveness of the *E. histolytica *genome provide a challenge for short-read mapping, yet we were able to define putative single nucleotide polymorphisms in a large portion of the genome. The results suggest a rather low level of single nucleotide diversity, although genes and gene families with putative roles in virulence are among the more polymorphic genes. We did observe large differences in coverage depth among genes, indicating differences in gene copy number between genomes. We found evidence indicating that recombination has occurred in the history of the sequenced genomes, suggesting that *E. histolytica *may reproduce sexually.

**Conclusions:**

*E. histolytica *displays a relatively low level of nucleotide diversity across its genome. However, large differences in gene family content and gene copy number are seen among the sequenced genomes. The pattern of polymorphism indicates that *E. histolytica *reproduces sexually, or has done so in the past, which has previously been suggested but not proven.

## Background

*Entamoeba histolytica *is a parasite of the human large intestine, commonly contracted by ingesting contaminated water or food. The parasite has a two stage life-cycle consisting of a cyst, the infective stage outside of the host, and a trophozoite, the reproductive stage within the host. Invasive amoebiasis results when trophozoites attack the gut wall, leading to diarrhoea, dysentery and in some cases dissemination to organs (commonly the liver) where abscesses result [1]. If untreated, amoebic liver abscess can be fatal.

*E. histolytica *infection is endemic in many parts of the world where sanitation infrastructure is poor; elsewhere infection tends to be restricted to certain at-risk groups such as residents in institutions for the mentally handicapped and men who have sex with men [2]. In endemic areas, exposure can be extremely high: an annual incidence of 40% was estimated among children in an urban slum in Bangladesh [3]. The global prevalence of infection was estimated to be approximately 10% of the world's population in the 1980s [4]. Of these, approximately 90% were estimated to be asymptomatic carriers while 10% developed invasive amoebiasis, leading to 100,000 deaths per year [4]. This estimate was made prior to the redescription in 1993 of *E. histolytica *as two separate species, *E. histolytica *and *E. dispar *[5]. *E. histolytica *causes invasive amoebiasis while *E. dispar *is avirulent, and it was thought that *E. dispar *infection might account for the predominance of asymptomatic carriers. However, even considering only *E. histolytica *infections, invasive amoebiasis appears still to be a relatively rare outcome of infection.

Understanding the nature of amoebic virulence motivates a substantial body of research. Several lines of evidence suggest that genetic factors of the parasite affect its virulence: the morphologically identical but genetically distinct *E. dispar *appears to be avirulent; distinct *E. histolytica *genotypes have been detected in liver abscess and feces from the same infected person [6]; different endemic populations show different rates of liver abscess [7]; a short-term transmission chain of virulent parasites has been reported [8].

The *E. histolytica *genome has been sequenced to a draft level with 12.5-fold coverage genome assembly consisting of approximately 24 megabases in 888 scaffolds [9]. Subsequent reassembly with additional data produced a genome assembly of approximately 21 megabases in 1,496 scaffolds [10]. The ploidy of *E. histolytica *is uncertain though some studies suggest that it may be tetraploid [11]. As part of the effort to identify genetic determinants of virulence and to gain an understanding of the diversity of the parasite, we re-sequenced the complete genomes of a number of *E. histolytica *strains. We show that single nucleotide diversity is relatively low among these parasites, but that gene copy number variation may be a significant contributor to genome diversity. We also provide evidence that *E. histolytica *may reproduce sexually and define a set of high quality SNPs that could be used as markers for genotyping *E. histolytica *isolates.

## Results and discussion

### Sequencing and mapping of *Entamoeba *strains to the HM-1:IMSS reference genome

Eight strains of *E. histolytica *derived from infected humans and adapted to axenic laboratory culture, as well as two clones of different virulence phenotypes derived from the axenic strain HM-1:IMSS, were sequenced and mapped to the published genome sequence of the HM-1:IMSS strain. Details of the origins of each strain are shown in Table 1. All sequence data were submitted to the European Nucleotide Archive under the following accessions: ERS132616 (HM-1A); ERS132617 (HM-1B); ERS132618 (Rahman); ERS132619 (2592100); ERS132620 (PVBM08B); ERS132621 (PVBM08F); ERS132622 (IULA:1092:1); ERS132623 (HK-9); ERS132624 (MS84-1373); ERS132625 (MS27-5030).

**Table 1:** *Entamoeba histolytica *strains sequenced in this study

Strain	Origin	Date	Symptoms
HM-1A	Mexico	1967	Intestinal amoebiasis
HM-1B	Mexico	1967	Intestinal amoebiasis
Rahman	UK^a^	1964	Asymptomatic
2592100	Bangladesh	2005	Intestinal amoebiasis
PVBM08B	Italy	2007	Intestinal amoebiasis
PVBM08F	Italy	2007	Intestinal amoebiasis
IULA:1092:1	Venezuela	1992	Intestinal amoebiasis
HK-9	Korea	1951	Intestinal amoebiasis
MS84-1373	Bangladesh	2006	Asymptomatic
MS27-5030	Bangladesh	2006	Asymptomatic

^a^Isolated from a sailor, ultimate origin unknown.

The *E. histolytica *genome is known to be very A + T rich (approximately 75% A + T) and repetitive, which both make sequence alignment difficult. In order to assess this repetitiveness and estimate how much unique mapping we might expect from short sequencing reads, we made a set of 50 bp reads from the reference genome and mapped them to the reference: 72.36% aligned to only one location, 12.46% aligned to two different locations, 10.83% aligned to between 3 and 10 locations and 4.35% aligned to more than 10 locations. As reads were derived from the reference genome, these proportions relate directly to proportions of the genome, so we expected up to 14 megabases of sequence to be unique enough for unique alignment to 50 bp reads. This shows that the majority of the reference genome is unique enough for accurate alignment of 50 bp reads. When we aligned the read libraries, we saw that approximately 75% of reads that aligned uniquely and approximately 13 megabases of the reference genome were covered by uniquely aligned reads, broadly consistent with our expectations.

Table 2 shows statistics relating to read alignment and SNP calling in the strains. Coverage of the genome was generally much better across coding sequences than across intergenic regions and the proportion of each gene covered was generally high (Additional file 1). The poorer coverage of intergenic regions could be due to their repetitiveness and their more extreme nucleotide composition bias towards A and T, as sequencing libraries can be biased toward more balanced nucleotide compositions.

**Table 2:** Alignment and SNP-calling statistics

Strain	Reads^a^	c50, c95 cov^b^	SNP-able sites^c^	SNP	Hom^d^	Het^e^	Divergence (hom only)^f^
HM-1A	13,743,406	35,141	10,012,951	2,217	229	1,988	0.22 (0.02)
HM-1B	9,586,924	26,95	9,819,882	1,995	220	1,775	0.20 (0.02)
Rahman	19,498,380	32,198	9,817,503	6,889	3,767	3,122	0.70 (0.38)
2592100	13,560,609	26,127	10,025,805	6,788	3,128	3,660	0.68 (0.31)
PVBM08B	17,627,870	36,172	10,335,217	7,999	4,225	3,774	0.77 (0.41)
PVBM08F	8,436,907	19,65	10,253,328	6,602	3,613	2,989	0.64 (0.35)
IULA:1092:1	19,041,335	48,155	11,934,434	10,014	4,897	5,117	0.84 (0.41)
HK-9	21,193,087	41,202	10,678,584	9,155	4,428	4,727	0.86 (0.41)
MS84-1373	21,479,273	51,209	10,308,534	8,373	4,027	4,346	0.81 (0.39)
MS27-5030	20,403,218	47,225	8,731,329	7,001	3,302	3,699	0.80 (0.38)

^a^'Reads' = the number of uniquely aligned reads. ^b^'c50, c95 cov' = the 50th and 95th centile of coverage depth. ^c^'SNP-able sites' = positions passing criteria for allowing a SNP to be called (see Materials and methods). ^d^'Hom' = homozygous SNP. ^e^'Het' = heterozygous SNP. ^f^'Divergence (hom only)' = divergence from reference genome (divergence calculated using only homozygous SNPs).

SNP calling was carried out, using thresholds for minimum and maximum coverage and minimum mapping quality. The number of sites passing these thresholds ranged from approximately 9 to 14 megabases. At these positions, bases were predicted and SNPs called (Additional file 2). SNP calls for heterozygotes will be affected by the unknown ploidy (a ploidy of 4 was assumed based on the estimate of Willhoeft and Tannich [11]). At least 89.55% of the homozygous SNPs in the Rahman strain were also called as variants when sequenced independently using different sequencing technology (454 sequencing; Additional file 3). However, as few as 43.02% of the heterozygous SNPs were similarly validated. It is difficult to ascertain whether the unvalidated SNPs are false positives or are due to the lower coverage of the 454 sequencing. Due to the greater uncertainty associated with heterozygous SNP calls, homozygous SNP calls showing inter-strain variation and a base call in every strain were tabulated as a set of 'high quality candidate markers' (Additional file 4). These SNPs were used to infer a phylogeny of sequenced strains and to test for evidence of meiotic recombination.

### Genealogy of *E. histolytica *strains

**Figure 1:**
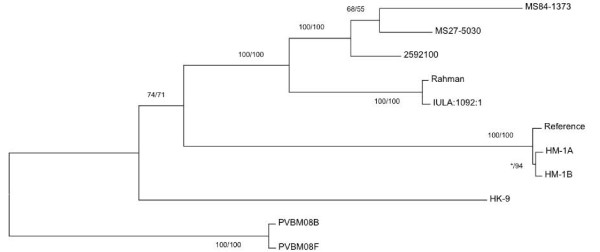
**Relationships among sequenced *E. histolytica *strains**. Relationships were estimated using 3,696 polymorphic sites and two phylogenetic approaches, distance-based analysis (neighbor-joining) and maximum likelihood (ML), both as implemented in MEGA 5 [52]. ML used the General Time Reversible model of evolution, selected as best using the model testing function in MEGA 5, while neighbor-joining used the maximum composite likelihood model. Statistical support for distance and ML trees was evaluated using bootstrapping (1,000 replicates). The ML tree is shown. The tree is rooted by comparison with orthologous positions in *E. dispar *(data not shown) along the branch to the PVBM08B and PVBM08F strains. Bootstrap values from both types of phylogenetic analysis are shown in the following order: Distance/ML. An asterisk indicates where the bootstrap value is under 50%.

Figure 1 shows the relationship among the sequenced strains. In this phylogeny the three strains derived from Bangladesh cluster together. The HM-1:IMSS-derived clones HM-1A and HM-1B cluster very closely with HM-1:IMSS. The pair of strains isolated from feces (PVBM08F) and from colonic biopsy (PVBM08B) from the same patient also cluster very closely together. The geographical origin of this pair of isolates is not known, but the patient from whom they were isolated had visited both Colombia and Liberia prior to the strains being isolated. These strains form an outgroup to the other strains when the phylogeny is rooted relative to *E. dispar*. HK-9 (isolated in Korea) appears to be rather different to the other strains. Rahman and IULA:1092:1 cluster very closely together. Rahman was originally isolated from a sailor in the UK, so its ultimate geographical origin is unknown. IULA:1092:1 was isolated in Venezuela.

### Evidence of meiotic recombination among *E. histolytica *strains

**Figure 2:**
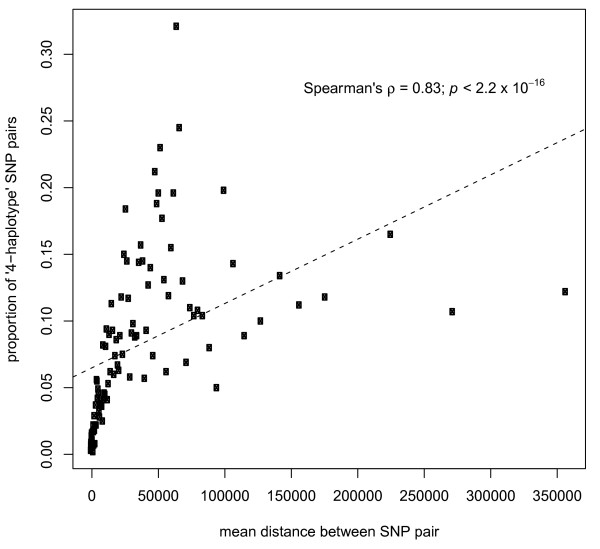
**Evidence of historical recombination among *E. histolytica***. The proportion of 100,000 randomly sampled pairs of polymorphic sites showing 4 haplotypes calculated for 100 distance intervals, plotted against the median distance for each interval. The positive correlation is highly significant, Spearman's rho = 0.83 (*P *< 2.2 × 10^-16^).

Under the infinite sites model of evolution, nucleotide sites mutate only once in the evolutionary history of a sample of related sequences. Given this, the occurrence of four haplotypes due to recombination should be more common for distant pairs of polymorphic positions as the probability of a recombination event increases with distance. Random sampling of pairs of polymorphic sites on the same reference genome scaffold showed that the proportion showing four haplotypes was significantly positively correlated with distance (Figure 2; Spearman's rho = 0.83, *P *< 2.2 × 10^-16^). The average distance between sites forming two haplotypes (indicating linkage disequilibrium among sites) was 18,485 bp, between sites forming three haplotypes was 46,644 bp and between sites forming four haplotypes (indicating a recombination event) was 60,176 bp. Among polymorphic sites in the same genomic scaffold (that is, physically linked in the genome), the proportion of two-haplotype pairs was 23.2%, three-haplotype pairs 68.3% and four-haplotype pairs 8.5%. In contrast, among polymorphic sites in different genomic scaffolds, the proportion of two-haplotype pairs was markedly lower (7.2%), while the proportions of three- and four-haplotype pairs were higher (81.1% and 11.7%, respectively), reflecting a reduction in linkage disequilibrium among sites on different chromosomes. Physically unlinked sites would be expected to remain in linkage disequilibrium in strictly asexual lineages. In order to support our inference of recombination in the history of the sequenced strains, we simulated the expected effects of recombination and of the analogous process of gene conversion upon the test. The results showed that, as the rate of recombination increases, the proportion of pairs of SNP showing all four possible haplotypes increases with distance (with a significant positive correlation). However, when gene conversion is modeled, the proportion of 'four-haplotype pairs' is not significantly positively correlated with distance (Additional file 3). Taken together, these results strongly suggest that sexual reassortment of chromosomes and meiotic recombination have occurred in the history of these genomes. Genetic data from a single, putatively panmictic, population will be required to confirm this result and to estimate the frequency of recombination events.

### SNPs among *E. histolytica *strains

Overall genetic diversity appears to be rather low among these genomes. For these data it is difficult to make an estimate of diversity such as the average pairwise divergence because both the ploidy and the phase of variant bases are unknown. Also, all data are mapped to a single reference genome, so comparisons to each other may not account for differences in genome coverage. Therefore, we estimated the divergence of each genome from the HM-1:IMSS reference genome as the proportion of sites that could be called as SNPs that were called as SNPs. We used both the number of putative homozygous SNPs and the total number of both homozygous and heterozygous SNPs to estimate a lower and an upper boundary for divergence. The estimates are shown in Table 2. Divergence estimates counting only putative homozygous positions were between 0.3120 and 0.4147 SNPs per kilobase, while estimates counting both putative homozygous and heterozygous positions were between 0.6439 and 0.8573 SNPs per kilobase.

All SNP calls estimated for each strain were tabulated and can be viewed in Additional file 2. In order to identify polymorphic genes that may be under diversifying selection, the numbers of nonsynonymous and synonymous homozygous SNPs per gene (compared to the HM-1:IMSS reference genome) were counted and tabulated in Additional file 5. Putative nonsense mutations in genes are tabulated in Additional file 6. In these Additional files, only homozygous variants were counted in order to reduce the chance of counting false heterozygous SNPs caused by alignment of paralogous sequence. Genes with five or more nonsynonymous homozygous SNPs in one or more strain are tabulated in Additional file 7. This rather crude method was used instead of estimating dN (nonsynonymous divergence per nonsynonymous site) and dS (synonymous divergence per synonymous site), despite its bias towards longer genes and genes with no closely related paralogues (that is, genes with a greater proportion of unique coverage), because the small number of variant positions per gene makes dN and dS estimates very sensitive to small stochastic differences in the number of variants and not robust. Fifty-three genes contained five or more homozygous nonsynonymous SNPs relative to HM-1:IMSS. Among these genes are several encoding proteins involved in host-parasite interactions, including the intermediate chains of the galactose/N-acetyl-D-galactosamine-inhibitable lectin (Gal/GalNAc lectin) *igl1 *(EHI_006980) and *igl2 *(EHI_065330), which were divergent in the HK-9 strain, the light chain Gal/GalNAc lectin gene *lgl *(EHI_035690), which was divergent in the Rahman and IULA:1092:1 strains, and three serine-threonine-isoleucine-rich protein (STIRP) genes (EHI_073630, EHI_004340 and EHI_012330). The Gal/GalNAc lectin complex is a major virulence factor expressed on the surface of the trophozoite that has multiple functions, including binding to the mucus layer and to epithelial cells and involvement in host cell killing [12]. STIRPs are putative trophozoite surface-expressed proteins, some or all of which bind to host epithelial cells and cause cell death [13].

**Figure 3:**
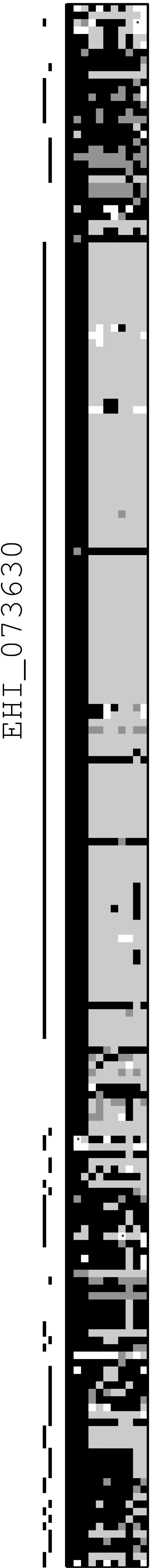
**The pattern of polymorphism across scaffold DS571171**. Polymorphic sites on scaffold DS571171 are represented by shaded squares: black for bases that match the HM-1:IMSS reference; light grey for bases that differ from the reference; dark grey for heterozygous positions; white for positions where no base was assigned. Three positions (marked with asterisks) had an additional variant base in one strain. Each row represents a strain in the following order from top to bottom: reference sequence (HM-1:IMSS), HM-1A, HM-1B, Rahman, 2592100, PVBM08B, PVBM08F, IULA:1092:1, HK-9, MS84-1373, MS27-5030. Sites in coding sequences are marked with black lines above the figure.

The gene that displayed most polymorphism, the STIRP gene EHI_073630, lay on the HM-1:IMSS scaffold DS571171. The gene is annotated as a STIRP, with predicted amino-terminal signal peptide and a carboxy-terminal transmembrane domain, but is sufficiently distantly related to other described STIRPs [13] that reads aligned uniquely across its entire length, unlike in other STIRP genes (data not shown). Across scaffold DS571171, the majority of polymorphic sites were in this gene and were homozygous in each strain. In addition, HM-1:IMSS and its derivatives HM-1A and HM-1B displayed a haplotype that differed greatly from the other strains, which were generally similar to each other. This pattern of polymorphism appears to break down outside of the gene (Figure 3). The HM-1-like allele differs from the others by 27 to 34 homozygous synonymous SNPs and 48 to 60 homozygous nonsynonymous SNPs across its 15,210 bp. Given an estimated 1,896 theoretically synonymous and 13,311 theoretically nonsynonymous sites, the estimated divergence values are dS = 0.014 to 0.017 and dN = 0.0036 to 0.0045 (dN/dS = 0.20 to 0.32). The relatively high value of dS (14 to 17 variants per kilobase) suggests that the alleles my have diverged a long time ago, consistent with a pattern of long-term allelic dimorphism as seen in some *Plasmodium *genes encoding surface-expressed proteins that interact with host cells [14,15].

### Gene copy number variation among *E. histolytica *strains

**Figure 4:**
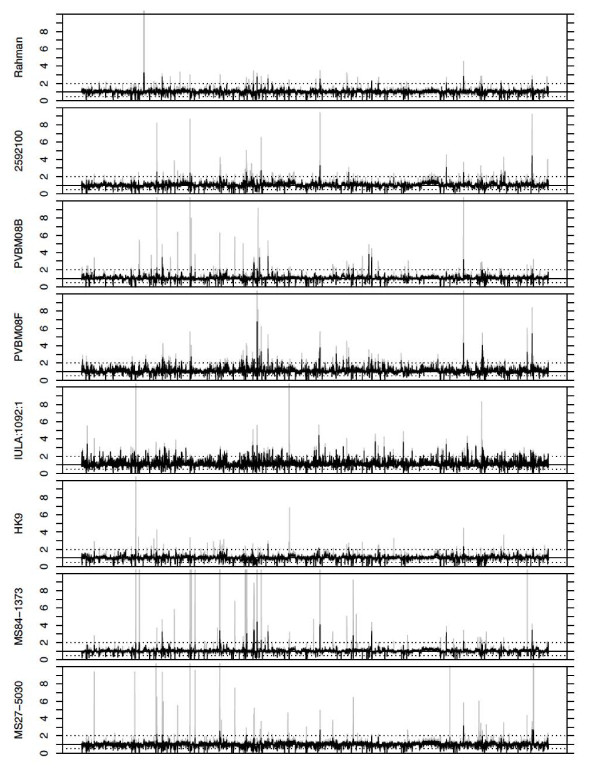
**Copy number variation among *E. histolytica *genes**. Coverage depth of a set of 6,228 *E. histolytica *genes relative to HM-1:IMSS in eight sequenced strains. The y-axis displays the ratio of a strain's RPKM to the average RPKM of HM-1A and HM-1B. The x-axis represents 6,228 annotated *E. histolytica *genes ordered by their amoebaDB gene ID. Ratios greater than 10 were truncated for display purposes. Grey bars represent results for all uniquely aligned reads, black bars for uniquely aligned reads with unique start positions on the reference genome (see Materials and methods).

Another source of genomic diversity in addition to SNPs is variation in the number of paralogous copies of genes and the content of gene families. To analyze this, depth of coverage of genes was compared between strains. While each strain showed a proportion of genes with high coverage, these were not the same genes in all strains. Figure 4 shows that strains varied considerably in the copy number of different genes, even putative close relatives such as PVBM0B and PVBM0F. This pattern remained generally consistent when all uniquely mapped reads were considered or only uniquely mapped reads with unique start positions were considered, although the signal was reduced overall and for some genes was lost.

Genes with coverage depth more than twofold the average of HM-1A and HM-1B in one or more strain were designated putative high copy number genes (Additional file 8). Putative missing genes (reads per kilobase per million mapped reads (RPKM) < 1 and < 50% of the average of HM-1A and HM-1B) were also tabulated (Additional file 9). Among the putative high copy number genes in one or more strain were genes encoding ribosomal proteins (EHI_081320, EHI_104660, EHI_108500, EHI_124300, EHI_124330, EHI_137870, EHI_152080, EHI_152570, EHI_152610, EHI_153070, EHI_179980, EHI_181560, EHI_182920, EHI_193330, EHI_199970). Also represented were members of large gene families encoding AIG1 (avirulence induced gene 1)-like proteins (EHI_022500, EHI_079610, EHI_115170, EHI_126560, EHI_144270, EHI_157260, EHI_176590, EHI_195250, EHI_195260), BspA (*Bacteroides forsythus *surface protein A)-like leucine rich repeat-containing proteins (EHI_002120, EHI_005660, EHI_008340, EHI_049160, EHI_094080, EHI_102380, EHI_113190, EHI_123820, EHI_137910, EHI_147680, EHI_163960, EHI_189090, EHI_191510, EHI_192600) and ariel family proteins (EHI_028430, EHI_057430, EHI_080200). Among the genes absent from different strains were genes on a duplicated genome region previously described in HM-1:IMSS [10], indicating variation in the copy number of these duplications among strains. Many putative missing genes were unannotated, and a proportion of them may be incorrect gene models. The results suggest that strains vary in their gene family content and that duplication and loss of members of these large gene families is a dynamic process.

**Figure 5:**
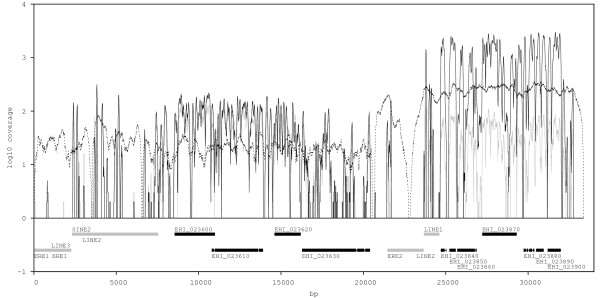
**Segmental duplication in the *E. histolytica *Rahman strain**. High coverage across a region of scaffold DS571330 in the Rahman genome is confirmed by independently generated sequencing libraries (SOLiD and 454). The region encompasses seven genes and is flanked by repetitive elements. The black line represents coverage from Rahman SOLiD sequencing library, the dashed line represents coverage from Rahman 454 sequencing library, the grey line represents coverage from strain HM-1B SOLiD sequencing library, and is shown as a control. Coverage depth is displayed on a log scale in order to show both SOLiD and 454 data (where many fewer reads are generated) on the same plot.

A region of high coverage occurring only in the Rahman strain and spanning several genes, encoding 60S ribosomal protein L38 (EHI_023840), a hypothetical protein (EHI_023850), protein kinase domain containing protein (EHI_023860), WD domain containing protein (EHI_023870), ubiquitin-conjugating enzyme family protein (EHI_023880), nuclear movement protein (EHI_023890) and hypothetical protein (EHI_023900), is likely to represent a segmental duplication similar to those described in HM-1:IMSS [10]. Data from an independently generated sequencing library of the Rahman strain sequenced by 454 sequencing (manuscript in preparation) confirms this putative segmental duplication (Figure 5). The duplicated genome region occurs adjacent to a repetitive element (*Entamoeba *repeat element 2, ERE2) and long interspersed nuclear elements (LINE1 and LINE2), which also mark a break in synteny between *E. histolytica *and *E. dispar *(data not shown). An approximate estimate of the number of copies of the duplicated region was made by comparing coverage depth in Rahman to that in HM-1B at sites in the unduplicated region (positions 1 to 21,000) and the duplicated region (positions 21,000 to 33,380). In the unduplicated region, the median ratio of coverage in Rahman to HM-1B was 1.1 and the 25th and 75th centiles were 0.7 and 1.6. In the duplicated region the median was 25.0 and the 25th and 75th centiles were 12.8 and 34.8. A similar putative segmental duplication in MS84-1373 spans a number of genes encoding unknown proteins (EHI_072460, EHI_072470, EHI_072480, EHI_072490, EHI_072500, EHI_072510, EHI_072520, EHI_072530).

## Conclusions

We report the genome-wide diversity of the enteric parasite *E. histolytica*. Overall, diversity among the sequenced genomes was rather low: divergence from the reference genome was between 0.312 and 0.857 SNPs per kilobase, depending on whether heterozygous SNPs were counted or not. An analogous measure of genomic diversity in *Plasmodium falciparum *estimated divergence from the reference strain 3D7 of a clinical isolate from Ghana (divergence = 1.31 SNPs per kilobase); of a strain (IT) derived from central America (divergence = 1.10 SNPs per kilobase); and divergence between *P. falciparum *3D7 and the related species *Plasmodium reichenowi *of 20.30 SNPs per kilobase [16]. Previous studies of genetic diversity among *E. histolytica *isolates have produced conflicting results. In general, studies using repetitive DNA as markers of diversity, such as the serine-rich *E. histolytica *protein (SREHP) gene and the short tandem repeats separating tRNA genes, have reported high diversity among genomes [17-21]. In contrast, studies of SNPs, though few in number, have reported minimal diversity [22-24]. Our results agree with the latter studies, suggesting that SNP is low in *E. histolytica*. However, our results also suggest that recombination and reassortment of alleles may have shuffled these alleles to create new haplotypes. Therefore, recombination and reassortment may be relatively more important than point mutation in generating diversity among parasite lineages.

The highly polymorphic STIRP gene EHI_073630 showed a pattern of polymorphism consistent with allelic dimorphism, as described in *P. falciparum*. It is difficult to say with certainty that the gene is truly dimorphic since it is a member of a gene family so it is possible that the aligned sequences are paralogous rather than allelic. However, the alignment clearly shows that the HM-1-like form of the gene is present in HM-1A and HM-1B and not present in the other strains. The causes and significance of allelic dimorphism in *P. falciparum *are not fully understood, but several genes that show this pattern of polymorphism (merozoite surface proteins MSP-1 [14], MSP-2 [25,26], MSP-3 [27], and MSP-6 [28], and erythrocyte binding antigen EBA-175 [29]) are involved in host cell invasion. Immune recognition of some of these proteins has been associated with protection [30-33], and a proportion of antibodies may recognize allele-specific epitopes [31,33,34]. Whether any of this applies to the *E. histolytica *gene EHI_073630 remains to be seen. More extensive sampling of this region of the genome from more parasite isolates might indicate whether or not EHI_073630 is truly dimorphic and if the HM-1-like form of the gene is geographically restricted to Mexico, or segregates in other populations.

Sexual reproduction has not been observed in *Entamoeba*. However, since the publication of the genome of the *E. histolytica *HM-1:IMSS strain showed that it possesses the majority of a set of genes involved in meiosis, it has been suggested that *E. histolytica *may reproduce sexually [9,35,36]. Gene conversion, a process involving homologous recombination, appears to have occurred among paralogous genes in the *E. histolytica *and *E. dispar *lineages [37]. The evidence from patterns of polymorphism presented here suggests that historical recombination events have broken up linkage between polymorphic sites and linkage has declined with increasing distance between sites on the same chromosome, consistent with the occurrence of meiotic recombination. Taken together, these lines of evidence strongly suggest that sexual reproduction has occurred in *E. histolytica *and adds it to the list of sexual protozoal parasites previously believed to be clonal, such as species of *Leishmania *[38] and *Giardia *[39]. Whether sexual reproduction is common in parasite populations could be tested by genotyping large numbers of individuals from endemic populations and measuring linkage disequilibrium.

Large differences in the depth of coverage among genes, indicative of gene copy number variation, were seen among the sequenced genomes, suggesting a degree of plasticity and variation in gene family content. A number of examples of polymorphism in the copy number of segmental duplications of regions spanning multiple genes were seen, such as that seen on scaffold DS571330 that appears to have been duplicated many times in the Rahman strain. Segmental duplications previously described in the HM-1:IMSS genome [10] appear to be present in the HM-1A and HM-1B clones but to show differential copy number among the other sequenced genomes (Additional file 9). Genomic plasticity can be an important generator of diversity and may be an important mechanism of molecular evolution in *Entamoeba*. Genomic plasticity is an important form of genome diversity among *Leishmania *species and strains [40]. Extensive size variation among homologous chromosomes is seen in *Entamoeba *[11] and in other parasitic protozoa such as trypanosomes [41]. If we consider the Rahman duplication in scaffold DS571330, 25 duplications of a 12.4 kilobase region would add around 0.3 megabases to the chromosome containing scaffold DS571330, a significant contribution to chromosomal size variation among strains. It remains to be seen whether such plasticity is a feature of natural parasite lines or a phenomenon arising during strain axenization, which is known to affect genome content [42].

In conclusion, our results suggest that among *E. histolytica *parasites, genomic plasticity and recombination may be relatively more important mechanisms of generating genome diversity than single nucleotide mutation.

## Materials and methods

### Origins and growth of *E. histolytica *strains and extraction of genomic DNA

Several *E. histolytica *isolates were sequenced. Isolates PVBM08F, PVBM08B, 2592100, MS84-1373 and MS27-5030 were axenized specifically for genome sequencing. The other isolates studied here are long-established laboratory strains.

PVBM08F and PVBM08B represent two culture isolations from the same individual, from feces and an intestinal biopsy, respectively. The male patient had recently spent 2 months in Liberia and presented with bloody diarrhoea and mucus, consistent with amoebic dysentery, and was strongly seropositive for *E. histolytica*. The cultures were kindly provided by Dra. Simonetta Gatti, Laboratory of Parasitology, Foundation IRCCS Policlinico San Matteo, Pavia, Italy.

MS84-1373 and MS27-5030 were established using routine monthly samples from asymptomatic children that were part of a long-term study of amoebiasis in Mirpur, Dhaka in Bangladesh [43]. Isolate 2592100 was from a case of amoebic dysentery seen at a local hospital in Dhaka. These cultures were kindly provided by Dr Rashidul Haque, International Centre for Diarrhoeal Disease Research, Bangladesh.

All five cultures were established in Robinson's medium [44]; on arrival in London they were adapted to growth in LYSGM with 5% adult bovine serum [45]. Cultures were then axenized via monoxenic cultures in LYI-S-2 (liver extract, yeast extract, iron, serum growth medium) with 15% adult bovine serum and *Crithidia fasciculata *as an associate, as described [44].

To generate large amounts of genomic DNA for genome sequencing, parasites were cultured axenically in LYI-S-2. Cells were grown to a high density and harvested, usually after 72 hours of growth. Cell cultures were centrifuged and the culture medium removed, then cells were washed twice in phosphate-buffered saline before lysis in 300 μl of QIAGEN cell lysis buffer (QIAGEN, Crawley, UK). Genomic DNA was purified using a modified version of the CTAB extraction protocol described elsewhere [46,47], using two rounds of phenol:chloroform:isoamyl alcohol (25:24:1) extraction. DNA was re-suspended in nuclease-free water, ready for preparation of sequencing libraries.

### SOLiD library preparation and sequencing

Purified genomic DNA was used to prepare SOLiD™ fragment libraries, according to the manufacturer's protocols (Life Technologies, Foster City, CA, USA). Briefly, genomic DNA was fragmented by sonication on a Covaris S2 sonicator (Covaris Inc., Woburn, MA, USA) and end-repaired in preparation for P1 and P2 adaptor ligation. Adaptors were ligated and the samples size selected and amplified by standard PCR. DNA was bound to SOLiD™ P1 beads and amplified by emulsion PCR, followed by enrichment for templated beads. The DNA was 3' modified before deposition on the sequencing slide, ensuring attachment of the beads to the slide. All libraries were sequencing on SOLiD™ 4 sequencer (Life Technologies), to produce 50 bp reads.

### SOLiD read mapping

Sequence reads were mapped to the genome sequence of *E. histolytica *strain HM-1:IMSS using the Burrows-Wheeler Aligner (BWA) mapping software [48]. The reference sequence matched that in data release 1.3 of the AmoebaDB database [49]; its 1,496 scaffolds contained 20,734,772 bases (excluding unknown bases represented by 'N'). Mapping parameters allowed up to four colorspace mismatches (that is, equivalent to up to two SNPs) per 48 bp read (after trimming off 5' and 3' terminal color calls), which represents up to approximately 4.2% divergence from the reference. The alignments were filtered to retain only uniquely aligned reads. These alignments were used for all further analyses.

### Analysis of repetitiveness of the reference genome

Non-unique mapping to the reference genome will lead to gaps in coverage of the genome. In order to assess mapping to the reference, the relative repetitiveness and uniqueness of the reference genome was estimated. The reference sequence was used to generate 50 bp sequence fragments representing the entire sequence, each offset from the last by 1 bp. These fragments were mapped to the reference genome using the Bowtie v0.12.1 short read mapper [50], allowing 0 mismatches. The number of times each fragment exactly matched a genomic region was recorded and the proportions of reads mapped to 1, 2, 3 to 10, 11 to 100 and > 100 different positions were calculated.

### Analysis of SNP variants

Nucleotide variants between mapped strain and reference were called separately for each strain using the SAMtools pileup software [51]. Bases were called for each strain using the MAQ algorithm, assuming a ploidy of 4 [11]. Putative SNP positions were filtered to remove those with coverage < 5, coverage in the top 5% (to remove potential artifactual heterozygous SNPs caused by 'stacking-up' of paralogous sequence), SNP quality < 20 or mapping quality < 20 (SNP and mapping quality scores are phred-scaled: 20 represents 99% accuracy of the call) or within 5 bp of a putative insertion or deletion. The effect of varying these threshold parameters on the predicted number of SNPs (in the Rahman strain) was assessed (Additional file 3). An independently generated dataset of Roche 454 reads representing the Rahman genome (manuscript in preparation) was used to validate SNP calls from the SOLiD data (Additional file 3). These reads were mapped to the same reference genome as the SOLiD data. The mean coverage across SNPs was 24×. All SNP calls were tabulated and are displayed in Additional file 2. In order to define a subset of high confidence candidate SNP markers to estimate a phylogeny and to test for evidence of recombination among strains, 3,696 SNPs that varied between strains but were putatively homozygous within each strain, occurred in coding sequences and had a base called in every strain were designated 'high quality candidate marker' SNPs (Additional file 4).

### Estimating a phylogeny of strains

The 3,696 'high quality candidate marker' SNPs were used to estimate a phylogeny of the sequenced *E. histolytica *strains. *E. histolytica*-only phylogenies (based on 3,696 positions) were estimated using two phylogenetic approaches: distance-based analysis (neighbor-joining) and maximum likelihood (ML). Both methods were implemented in MEGA 5 [52]. ML used the General Time Reversible model of evolution, selected as best using the model testing function in MEGA 5, while neighbor-joining used the maximum composite likelihood model. Statistical support for distance and ML trees was evaluated using bootstrapping with 1,000 replicates.

In order to root these phylogenies, a set of *E. dispar *variants at orthologous positions was defined. This was done by aligning regions flanking *E. histolytica *SNP positions (100 bp on either side) to the *E. dispar *genome assembly using NUCmer [53] and filtering the alignments to remove any that were not unambiguous best alignments from both species. This resulted in a set of 1,897 positions at which an *E. dispar *base could be confidently determined. This set of positions was used to build a bootstrapped neighbor-joining phylogeny using programs from the PHYLIP software package [54], which placed the *E. dispar *branch along the branch linking PVBM08B and PVBM08F to the other *E. histolytica *strains. The *E. histolytica*-only phylogenies were rooted along this branch.

### Testing for evidence of recombination among lineages

The four-gametes (or four-haplotypes) test was applied to test for evidence of recombination during the evolutionary history of the *E. histolytica *strains. Under the infinite sites model of evolution, nucleotide sites mutate only once in the evolutionary history of a sample of related sequences, with no subsequent or back-mutation. Therefore, the maximum number of possible haplotypes formed by a pair of nucleotide sites in the absence of recombination is three. Four haplotypes can only be produced by a recombination event occurring between the two positions. Moreover, if recombination has happened, the occurrence of four haplotypes should increase in frequency with greater distance between variable nucleotide sites.

In order to detect this, 100,000 pairs of polymorphic 'high quality candidate marker' positions on the same scaffold (that is, putatively physically linked) were randomly sampled and the proportion of pairs showing four haplotypes was calculated for 100 distance intervals corresponding to centiles of the distance (each of 1,000 pairs of polymorphic sites). The proportion of pairs showing four haplotypes was plotted against the median distance for each interval. The correlation between this proportion and the distance between positions was tested by Spearman's rank correlation.

To strengthen the conclusion of meiotic recombination based on the results of the four-haplotypes test, we carried out simulations of samples undergoing recombination or gene conversion to compare the expected patterns of polymorphism. Simulations were carried out using Hudson's 'ms' software [55], which generates samples of sequence by coalescent simulation. In this case, two mutations were simulated (randomly assigned to branches of the coalescent tree) 100,000 times. Recombination was modeled by specifying a recombination parameter (4.Ne.r, where Ne is the effective population size and r is the per generation probability of a crossover occurring in the sequence). Gene conversion was modeled by specifying a parameter similar to the recombination parameter (4.Ne.f, where f is the per generation probability of a gene conversion event in the sequence) as well as the length of the 'converted' region. The proportion of four-haplotype SNP pairs per centile interval (that is, 1,000 simulations) was plotted.

### Analysis of gene copy number variation

In order to detect variation in gene copy number among strains (including duplicated and missing genes), read counts were estimated for each gene in each strain. These counts were normalized for different library sizes between strains and for different gene lengths by expressing each as RPKM. In order to detect copy number variation, each strain was compared to HM-1:IMSS (in this case, the average RPKM for the HM-1A and HM-1B strains). Genes where the average HM1 RPKM was very high (top 12.5%) or low (bottom 12.5%) were not considered in the analysis, to avoid calling genes as missing when the lack of unique mapping is actually due to repetitiveness, leaving a set of 6,228 genes. Genes with RPKM values two-fold higher or two-fold lower were considered to have a variable number of copies. In addition, putative missing genes had to have an RPKM value of < 1. The analysis was run both for the original alignments and for alignments filtered so that only reads with unique start positions were considered. This second analysis was done to remove the possibility that high coverage was due to PCR duplicates arising during sequencing library preparation. However, with short read fragment libraries such as these, and particularly across short genes with deep coverage, many non-unique start positions are expected by chance, so removing them will dampen the signal of a high copy number. Therefore, the results of both analyses were shown.

### Analysis of SNP polymorphism in gene coding sequences

SNPs in the coding regions of genes were classified as nonsynonymous (amino acid-altering) or synonymous (causing no change to the protein sequence) using custom perl scripts. Counts of the numbers of homozygous nonsynonymous and synonymous SNPs in polymorphic genes were tabulated (Additional file 5).

### Data availability

All sequence read data have been deposited in the EBI short read archive and are publicly available. All read data are labeled with the study accession number ERP001383 and individual samples are labeled with the following sample accession numbers: HM-1A, ERS132616; HM-1B, ERS132617; Rahman, ERS132618; 2592100, ERS132619; PVBM08B, ERS132620; PVBM08F, ERS132621; IULA:1092:1, ERS132622; HK-9, ERS132623; MS84-1373, ERS132624; MS27-5030, ERS132625.

## Abbreviations

bp: base pair; Gal/GalNAc-lectin: galactose/N-acetyl-D-galactosamine-inhibitable lectin; ML: maximum likelihood; MSP: merozoite surface protein; RPKM: reads per kilobase per million mapped reads; SNP: single nucleotide polymorphism; STIRP: serine: threonine and isoleucine rich protein.

## Competing interests

The authors declare that they have no competing interests.

## Authors' contributions

Experiments were conceived and designed by NH, SP, and CGC. Analyses were carried out by GDW. Cell cultures of HM-1A and HM-1B were grown and DNA isolated by IB and ET; all other strains were grown and genomic DNA isolated by CGC and GDW. DNA sequencing libraries were made, and sequencing carried out, by SK and PK. The manuscript was drafted by GDW, with contributions from all authors. All authors read and approved the final manuscript for publication.

## Supplementary Material

Additional file 1 **Coverage of genes in sequenced strains**. Histograms of the proportion covered of *E. histolytica *genes. The proportions of *E. histolytica *genes with 0 to 10%, 11 to 20%, 21 to 30%, 31 to 40%, 41 to 50%, 51 to 60%, 61 to 70%, 71 to 80%, 81 to 90% and 91 to 100% of their sequence covered are plotted. The majority of genes are well covered (91 to 100% of their length) by sequence libraries. Click here for file

Additional file 2 **Table of single nucleotide variants in *E. histolytica *strains relative to the HM-1:IMSS reference genome**. SNP calls relative to the HM-1:IMSS reference genome. Click here for file

Additional file 3 **Additional analyses carried out to test the effects of SNP calling parameters, verify called SNPs and to simulate the four-haplotypes test**. Additional analyses carried out to test the effects of varying the SNP calling parameters on the numbers of SNPs called in the Rahman strain, verify the called SNPs in Rahman strain by comparison with an independently generated dataset and to simulate the four-haplotypes test under models of varying recombination and gene conversion. Click here for file

Additional file 4 **Table of high quality candidate marker SNPs**. Table of high quality candidate markers: defined as SNPs that vary between strains but are homozygous within each strain, occur in coding sequences and have a base called in every strain sequenced. Click here for file

Additional file 5 **Table of polymorphic genes in *E. histolytica *strains relative to the HM-1:IMSS reference genome**. Counts of homozygous nonsynonymous and synonymous SNPs in polymorphic genes. Click here for file

Additional file 6 **Table of putative nonsense mutations in *E. histolytica *genes**. Table of SNPs causing putative nonsense mutations in *E. histolytica *strains. The table includes both homozygous and heterozygous SNPs. Click here for file

Additional file 7 **Highly polymorphic genes in *E. histolytica***. Table of genes with five or more homozygous nonsynonymous SNPs in one or more strains, with details of protein features. Click here for file

Additional file 8 **Genes showing deep coverage in one or more *E. histolytica *strains**. Table of genes (with RPKM values) with more than two-fold greater depth of coverage than the average of HM-1A and HM-1B in one or more strain, representing putative high copy number genes in that strain. Click here for file

Additional file 9 **Genes putatively missing from one or more *E. histolytica *strains**. Table of genes (with RPKM values) with RPKM < 1 and where this value is < 50% of the average of HM-1A and HM-1B, representing putative missing genes in that strain. Click here for file
