# Supplementary figures and images for: Genomic diversity of the human intestinal parasite Entamoeba histolytica
Source: Genome Biol. 2012 May 25;13(5):R38. doi: 10.1186/gb-2012-13-5-r38 (PMC3446291; doi:10.1186/gb-2012-13-5-r38)

**HM-1A**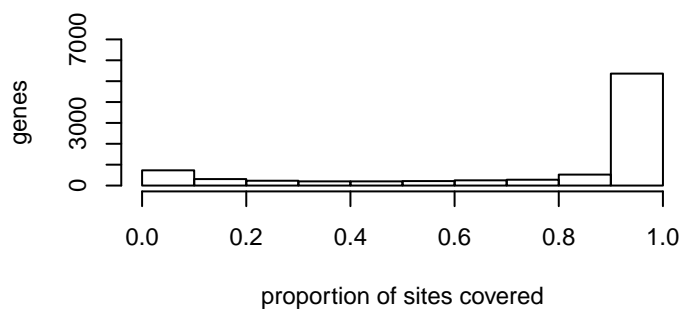**HM-1B**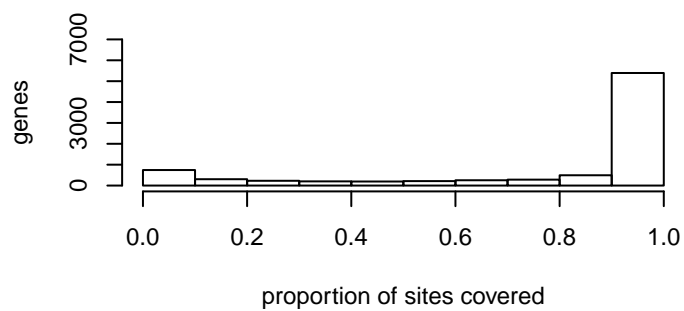**Rahman**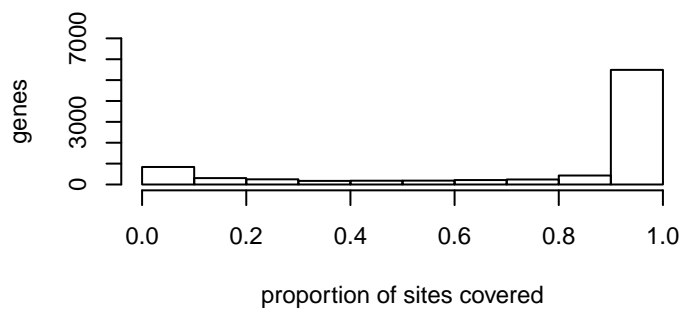**2592100**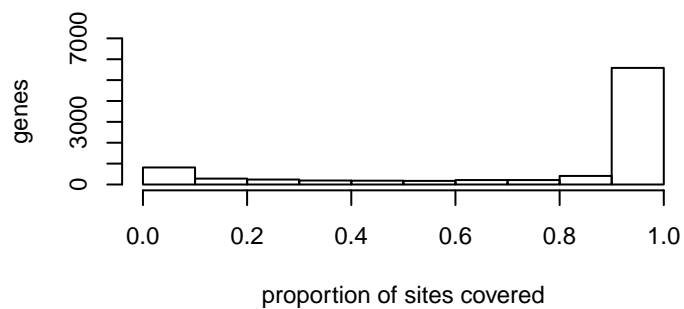**PVBM08B**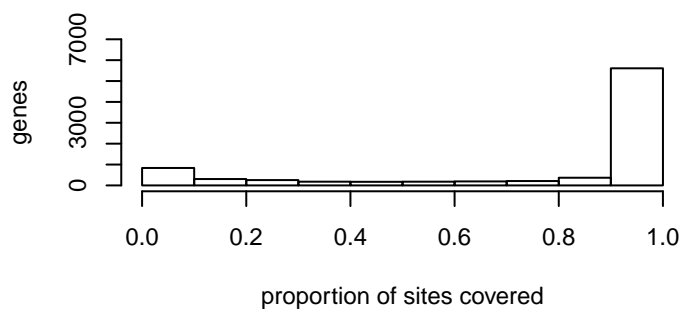**PVBM08F**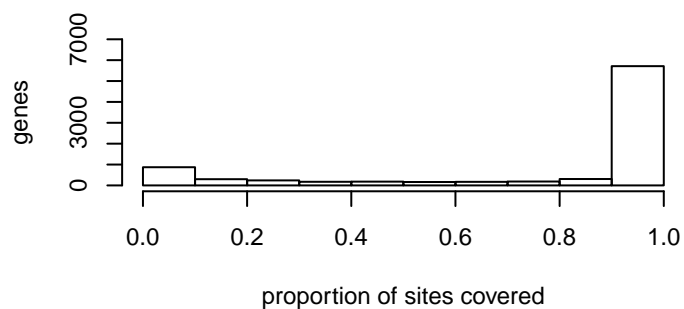**IULA:1092:1**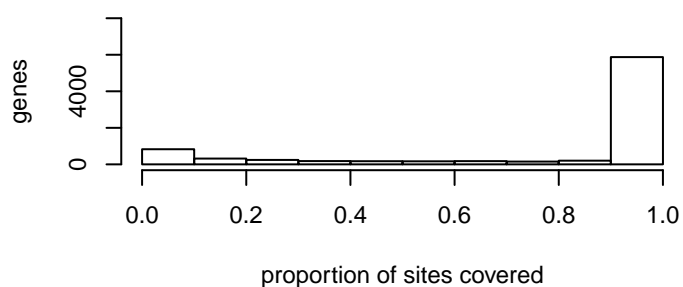**HK-9**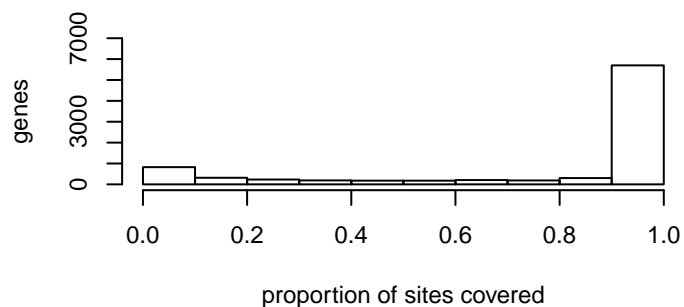**MS84-1373**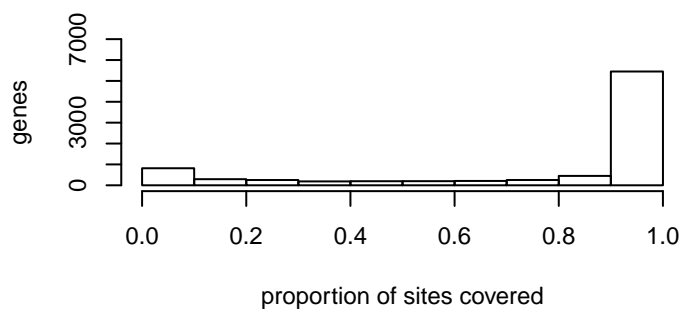**MS27-5030**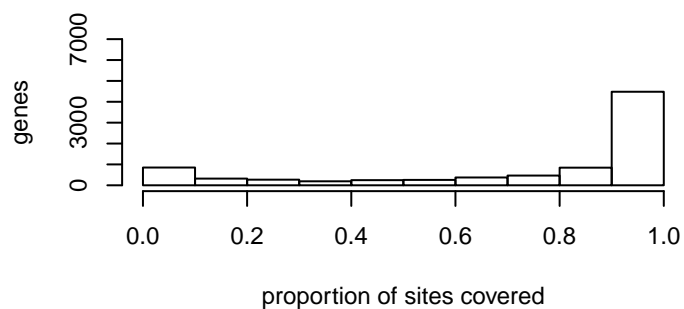

Supplement: Additional file 1 — Coverage of genes in sequenced strains. Histograms of the proportion covered of E. histolytica genes. The proportions of E. histolytica genes with 0 to 10%, 11 to 20%, 21 to 30%, 31 to 40%, 41 to 50%, 51 to 60%, 61 to 70%, 71 to 80%, 81 to 90% and 91 to 100% of their sequence covered are plotted. The majority of genes are well covered (91 to 100% of their length) by sequence libraries. [file gb-2012-13-5-r38-S1.PDF]
