# Supplementary material for: Genomic diversity of the human intestinal parasite Entamoeba histolytica
Source: Genome Biol. 2012 May 25;13(5):R38. doi: 10.1186/gb-2012-13-5-r38 (PMC3446291; doi:10.1186/gb-2012-13-5-r38)

Additional analyses

The effect of varying SNP filtering threshold parameters

The effect of varying threshold parameters on the predicted number of SNP was determined for the Rahman strain. The three parameters used to filter data from the samtools pileup programme were: SNP quality (“snpQ”, a measure of confidence that a non-reference allele occurs at a position); the root mean square of mapping quality (“RMSmapQ”, measure of confidence in the read alignment across a position); and depth of coverage at the position (“cov”). The threshold values used to call SNP in this study were: snpQ>=20; RMSmapQ>=20; coverage>=5. Each was varied (snpQ>=20, 25, 30, 35; RMSmapQ>=20, 25, 30, 35; coverage>=5, 6, 7, 8, 9, 10) and applied to the alignment for the *E. histolytica* Rahman strain. The total number of SNP and the numbers of homozygous and heterozygous SNP were recorded in the following table.

Table showing SNP counts under different threhold parameter values (data for Rahman strain).

| **snpQ** | **RMSmapQ** | **cov** | **SNP** | **hom** | **het** |
| --- | --- | --- | --- | --- | --- |
| 20 | 20 | 5 | 6884 | 3764 | 3120 |
| 20 | 20 | 6 | 6580 | 3510 | 3070 |
| 20 | 20 | 7 | 6276 | 3284 | 2992 |
| 20 | 20 | 8 | 6045 | 3121 | 2924 |
| 20 | 20 | 9 | 5807 | 2954 | 2853 |
| 20 | 20 | 10 | 5627 | 2833 | 2794 |
|  |  |  |  |  |  |
| 20 | 20 | 5 | 6884 | 3764 | 3120 |
| 25 | 20 | 5 | 6643 | 3746 | 2897 |
| 30 | 20 | 5 | 6393 | 3700 | 2693 |
| 35 | 20 | 5 | 5839 | 3313 | 2526 |
|  |  |  |  |  |  |
| 20 | 20 | 5 | 6884 | 3764 | 3120 |
| 20 | 25 | 5 | 6851 | 3734 | 3117 |
| 20 | 30 | 5 | 6249 | 3254 | 2995 |
| 20 | 35 | 5 | 3913 | 2116 | 1797 |

Verification of putative SNP

In order to assess the confidence of the SNP calls from the SOLiD sequence data, SNPs called in the Rahman strain alignment were compared to an independently generated set of reads for this strain, sequenced using the Roche 454 platform. These 454 reads are generally fewer and longer than SOLiD reads. The reads were mapped to the *E. histolytica* HM-1:IMSS reference genome (the same as used for the SOLiD read mapping) using the Newbler GSMapper software (Roche).

For 3767 putative homozygous SNP and 3122 putative heterozygous SNP called in the Rahman strain (SOLiD), 3685 (97.82 %) and 3078 (98.59 %) of the positions had a base called from the 454 mapping data.

Of the 3685 homozygous SOLiD SNP with a basecall in the 454 mapping, 3300 (89.55 %) were called as ‘high confidence differences’ in the 454 mapping (‘high confidence differences’ are positions where a non-reference allele is supported by at least 3 454 sequence reads, with at least 2 in one direction and 1 in the other). 3345 (90.77 %) of the SOLiD homozygous SNP were present in the ‘all differences’ class (positions with a non-reference allele, but with less stringent requirements than for ‘high confidence differences’).

However, of 3122 putative heterozygous SNP with a basecall in the 454 mapping, only 1343 (43.02 %) and 1435 (45.96 %) were represented among 454 ‘high confidence differences’ and ‘all differences’, respectively. This can be partially accounted for by a tendency for the lower coverage 454 alignment to miss heterozygous SNP. However, it also reduces our confidence in the heterozygous SNP calls from the SOLiD data. Based on this, we decided to consider only putative homozygous SNP for our analyses.

Modelling the 4-haploypes test to detect a signature of recombination with no recombination, recombination or gene conversion

In order to support our inference of recombination in the history of the sequenced strains, we simulated the expected effects of recombination and of the analogous process of gene conversion upon the test. Simulations were carried out using Hudson's 'ms' software (Hudson, 2002), which generates samples of sequence by coalescent simulation.  In this case, two mutations were simulated (randomly assigned to branches of the coalescent tree) 100,000 times.  Recombination was modelled by specifying a recombination parameter (4.Ne.r, where Ne is the effective population size and r is the per generation probability of a crossover occuring in the sequence).  Gene conversion was modelled by specifying a parameter similar to the recombinatin parameter (4.Ne.f, where f in the per generation probability of a gene conversion event in the sequence) as well as the length of the 'converted' region.  The proportion of 4-haplotype SNP pairs per centile interval (i.e. 1000 simulations) was plotted.

The results show that as the rate of recombination increases, the proportion of pairs of SNP showing all four possible haplotypes increases with distance (with a significant positive correlation). However, when gene conversion is modelled, the proportion of ‘4-haplotype pairs’ is not significantly positively correlated with distance. Results are shown in the following table and figure.

Table showing Spearman's rank correlation between proportion of 4-haplotype SNP pairs and distance between SNPs.

| **Recombination parameter** | **Gene conversion parameter** | **Spearman’s rho** | **P-value** |
| --- | --- | --- | --- |
| 1 | 0 | 0.885 | <2.2e-16 |
| 5 | 0 | 0.976 | <2.2e-16 |
| 10 | 0 | 0.977 | <2.2e-16 |
| 50 | 0 | 0.957 | <2.2e-16 |
| 100 | 0 | 0.940 | <2.2e-16 |
| 0 | 1 | -0.184 | 0.0668 |
| 0 | 5 | 0.158 | 0.1174 |
| 0 | 10 | -0.059 | 0.5581 |
| 0 | 50 | 0.018 | 0.8625 |
| 0 | 100 | 0.059 | 0.5629 |


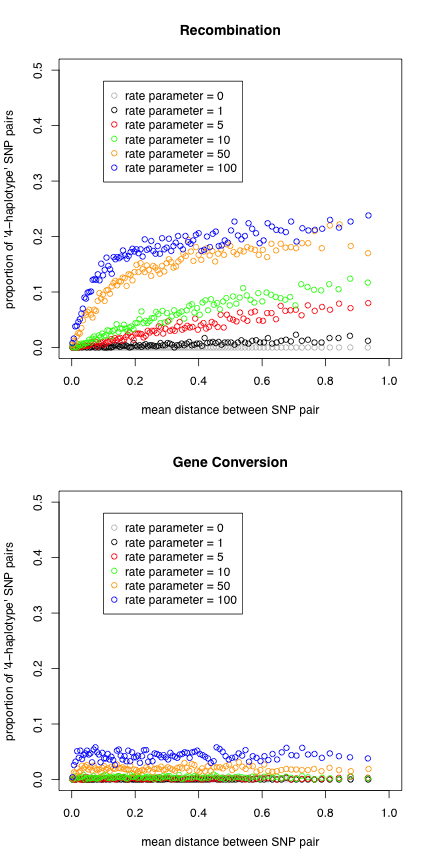

Supplement: Additional file 3 — Additional analyses carried out to test the effects of SNP calling parameters, verify called SNPs and to simulate the four-haplotypes test. Additional analyses carried out to test the effects of varying the SNP calling parameters on the numbers of SNPs called in the Rahman strain, verify the called SNPs in Rahman strain by comparison with an independently generated dataset and to simulate the four-haplotypes test under models of varying recombination and gene conversion. [file gb-2012-13-5-r38-S3.DOCX]
